# Characterization of Bioactive Compounds from Patchouli Extracted via Supercritical Carbon Dioxide (SC-CO_2_) Extraction

**DOI:** 10.3390/molecules27186025

**Published:** 2022-09-15

**Authors:** Syaifullah Muhammad, Abdul Khalil H. P. S., Shazlina Abd Hamid, Mohammed Danish, M. Marwan, Yunardi Yunardi, C. K. Abdullah, M. Faisal, Esam Bashir Yahya

**Affiliations:** 1 Chemical Engineering Department, Universitas Syiah Kuala, Banda Aceh 23111, Indonesia; 2 ARC-PUIPT Nilam Aceh, Universitas Syiah Kuala, Banda Aceh 23111, Indonesia; 3 Bioresource Technology Division, School of Industrial Technology, Universiti Sains Malaysia, Penang 11800, Malaysia; 4 Green Biopolymer, Coatings & Packaging Cluster, School of Industrial Technology, Universiti Sains Malaysia, Penang 11800, Malaysia; 5 Statistics Department, Universitas Syiah Kuala, Banda Aceh 23111, Indonesia; 6 Bioprocess Technology Division, School of Industrial Technology, Universiti Sains Malaysia, Penang 11800, Malaysia

**Keywords:** patchouli, supercritical carbon dioxide extraction, bioactive compounds, patchouli alcohol, qualitative characterization

## Abstract

Patchouli extracts and oils extracted from *Pogostemon cablin* are essential raw material for the perfume and cosmetics industries, in addition to being used as a natural additive for food flavoring. Steam distillation is a standard method used for plant extraction. However, this method causes thermal degradation of some essential components of the oil. In this study, patchouli was extracted with supercritical carbon dioxide (SC-CO_2_) under different conditions of pressure (10–30 MPa) and temperature (40–80 °C). The chemical components of the crude extracted oil and the functional group were characterized using gas chromatography-mass spectrometry (GC-MS) and Fourier Transform Infrared Spectroscopy (FT-IR). The extraction with supercritical carbon dioxide was shown to provide a higher yield (12.41%) at a pressure of 20 MPa and a temperature of 80 °C. Patchouli alcohol, Azulene, δ-Guaiene, and Seychellene are the main bioactive compounds that GC-MS results have identified. FTIR spectra showed alcohol, aldehyde, and aromatic ring bond stretching peaks. Extraction of patchouli with supercritical carbon dioxide provided a higher yield and a better quality of the crude patchouli oil.

## 1. Introduction

Patchouli (*Pogostemon cablin*) is an herbaceous plant native to South Asian countries that belongs to the Lamiaceae family [1]. It contains essential oil of great economic value [2]. Patchouli oil is ranked in the top 18 of 300 essential oils of commercial importance in the world [2]. Indonesia is the world’s largest producer of patchouli oil, accounting for more than 80% of the total annual production [3]. Patchouli oil has a high commercial potential in the international market due to its distinct flavor, smell, and biological activities [4]. Patchouli oil can be extracted from all parts of the plant, including roots, stems, branches, and leaves [1]. Generally, the oil content in leaves (2.5–5.0%) is higher compared to the essential oil content in roots, stems, and branches (0.4–0.5%), [5]. Patchouli plant contains a number of bioactive compounds, including terpenoids, phytosterols, flavonoids, organic acids, lignin, alkaloids, glycosides, alcohols and aldehydes [4].

Patchouli oil and extracts are well known for their pleasant flavoring, therapeutic, antibacterial, and antioxidant capabilities. As a result, they are widely used in the cosmetic and food industries [6]. Most significantly, patchouli oil acts as a fixative agent. A fixative agent is a substance that is used to reduce the rate of evaporation and can increase the stability of the mixture when added to more volatile components [7]. Its fixative properties give perfumes a strong and lasting character when combined with other essential oils [8]. Furthermore, the fixative characteristics of patchouli oil aid in reducing evaporation and improving firmness, making it useful in the production of soaps, fragrances, body lotions, and detergents. It is used in aromatherapy to calm nerves, reduce appetite, and alleviate depression and stress. Patchouli oil is also on the FDA (Food and Drug Administration) list of chemicals allowed for human consumption as a flavoring addition to natural foods [9]. Patchouli oil is widely used in the food industry as a flavoring ingredient in a variety of foods, including alcoholic and non-alcoholic beverages [1]. This oil is used at very low concentrations (2 mg/kg) to flavor foods, beverages, candy, and baked products [5].

Patchouli oil is often extracted using conventional methods, such as steam distillation [10]. However, this method has limitations such as long extraction time, low yield, and high-temperature effects [10,11]. High temperature is detrimental to heat-sensitive compounds present in essential oil, as it can cause a chemical alteration resulting in a different flavor and fragrance profile. In Indonesia, a typical steam distillation process requires 40 L of kerosene for an 8-h extraction, which recovers 2.2 to 2.8 kg of oil per 100 kg of patchouli leaves [10]. Therefore, an alternative extraction method with the minimum use of energy, solvent, and time is of considerable interest. In recent years, Supercritical Carbon Dioxide Extraction (SC-CO_2_) has gained popularity as an alternative to more conventional extraction methods because the dissolution power of supercritical fluids can be modified by adjusting the pressure and temperature conditions used [12,13]. The application of SC-CO_2_ to extract essential oils has gained great interest due to its low-temperature nature and solvent-free process [14]. The supercritical CO_2_ extractant is economical, inert, non-toxic, chemically stable, non-flammable, does not retain solvent residue in the extract and has a low critical temperature (31.1 °C) and a critical pressure (7.39 MPa) [15].

Patchouli oil extraction using SC-CO_2_ has been studied by some researchers [12,15]. Donelian et al. [16], for example, investigated the yield and chemical composition of essential oil extracted by SC-CO_2_ from patchouli leaves grown in Brazil. In addition to that, Liu et al. [12] investigated the yield of oil derived from Patchouli leaves and stems from China using SC-CO_2_ extraction. According to a literature survey, researchers are more likely to investigate the quality of patchouli oil after purification process than the crude patchouli oil that obtained after the extraction. Therefore, this study will characterize the crude Patchouli oil obtained from Patchouli plants including the leaves, branches, and stems to fill this research gap. At the same time, SC-CO2 extraction parameters were studied to increase the quality and enrich the patchouli alcohol content. The objective of this investigation was to observe the effect of the temperature and pressure of the SC-CO_2_ extraction on the yield and crude Patchouli oil. The quality of the crude patchouli oil composition was characterized by gas chromatography mass spectrometry (GC-MS). Fourier transform infrared (FTIR) spectroscopy was used to identify and provide the chemical functional group that is contained in the crude patchouli oil.

### Application of Patchouli Oil and Extracts

Patchouli oil and extracts have great commercial benefits and are widely used in perfumery, aromatherapy, pharmaceutical industries, and foods flavoring manufacturing [17]. The reason to emphasize the uses of natural products such as essential oil is their low toxicity profile [18].

The oily compounds in Patchouli are highly valued in perfumery and aromatherapy due to their dominant aromatic spicy fragrance. Patchoulol is the major constituent and is the primary component responsible for the typical Patchouli aroma. This compound also has the ability to blend well with other essential oils, providing a long-lasting base note in perfumes, preserving their natural freshness and fragrance [15]. It is widely used in perfumery and modern industrial products scented with odors, such as paper towels, laundry detergents, and air fresheners, as it provides a distinct and robust fragrance [8]. In aromatherapy, it is used to calm the nerves, control appetite, and release depression and stress.

Various therapeutic properties of patchouli oil include anti-inflammatory, antiseptic, astringent, diuretic sedative, and anti-mutagenic activities [19]. It is also effective for fungal and bacterial infections and is of great help for insect bites. On the skin, it is one of the active compounds that helps to stimulate the growth of new skin cells. In wound healing, it not only promotes faster healing, but also helps soothe the wound and scar skin and speed up the healing process [20]. Patchouli essential oil has been used as a remedy for skin problems such as acne, eczema, inflamed, and irritated skin. For hair, it was used for dandruff and to help oily hair with a marked aromatherapeutic response. For the nervous system, it helps to reduce tension, insomnia, and anxiety.

In addition to that, Patchouli oil and extracts are widely used in the flavoring industries and serve as an ingredient in many foods and beverages. It is also approved for human consumption and is safe to be a natural additive for food flavoring. Acting as an aromatizing agent, patchouli oil was used in major food products including alcoholic and nonalcoholic beverages, gelatin meat, meat products and frozen dairy dessert [16]. In the food industry, essential oils are used around 55–60% for flavoring purposes [1]. The worldwide market of essential oils was USD 7.6 billion in 2018 and is expected to reach USD 15.1 Billion by 2026 owing to its collective value in different sectors and the growing adoption in medicinal field of medicine [1]. Therefore, its commercial applications include the manufacturing of flavor, fragrance, and cosmeceuticals.

## 2. Results and Discussion

Statistical analysis of crude patchouli oil has been performed using a two-way analysis of variance (ANOVA) and the result is shown in Table 1. According to the ANOVA analysis, the significance level was set at 5% (0.05) and the confidence level at 95% (0.95). The *p*-values indicate that the pressure factor is significant, while the temperature factor is not significant due to a *p*-value greater than the significance level. In general, the pressure level is associated with a different yield of the SCCO_2_ process, and the temperature level is not associated with a different yield of the SCCO_2_ process. However, the extremely low *p*-value for the interaction between pressure and temperature showed that the process was statistically significant. Therefore, to extract a higher volume of crude patchouli oil, the pressure and temperature parameters should be considered. The effect of cross-interaction is probably to increase the pressure with constant temperature or increase the temperature with variable pressure to obtain a higher volume of crude patchouli oil.

The extraction method has a direct impact on the bioactive components. The high-temperature and-pressure extraction method degrades the heat-sensitive component of the extract. Therefore, it is important to study the components of oils extracted from various biomasses under different conditions. At different combinations of temperature and pressure, the bioactive components of the extract were identified and quantified by gas chromatography-mass spectroscopy (GC-MS), and functional groups in the bioactive component of the extract were explored through Fourier transform infrared (FTIR) spectroscopy studies.

### 2.1. Effect of Pressure and Temperature in SC-CO_2_ Extraction

**Figure 1:**
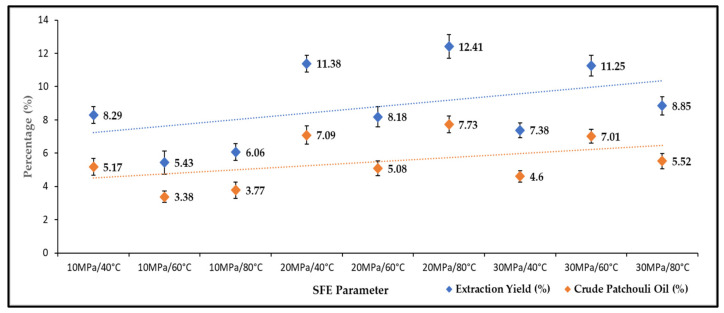
Extraction yields and crude patchouli extract percentages.

The effects of pressure and temperature on the yield of total oil extract were studied under varying operating pressures (10–40 MPa) and temperatures (40–80 °C). Figure 1 shows the effect of pressure and temperature on the extraction yield and production of crude patchouli extract. The extraction yield is the first extract collected after 60 min of the SC-CO_2_ extraction process. Meanwhile, crude patchouli oil is the densified oil (separation by centrifuge). The highest yield of SC-CO_2_ extraction reached 12.41 wt.% (wet sample), obtained at a temperature of 80 °C and a pressure of 20 MPa. While the lowest extraction yield (5.43 wt.%) was obtained at a pressure of 10 MPa and 60 °C temperature. Generally, with increasing pressure, the yield of patchouli crude extract increased. At 20 and 30 MPa, the oil extract yields were higher than 10 MPa pressure. The increase in pressure is believed to increase the percent extraction due to an apparent increase in SC-CO_2_ density [21]. This means that, if the density of SC-CO_2_ is sufficient to extract certain compounds, then higher percentages of extraction yields could be produced. The extraction efficiency of Patchouli with supercritical CO_2_ has been shown to increase with temperature and pressure. However, the pressure effect plays an important role compared to the temperature effects in extraction recovery. In Figure 1, it could be seen that most of the extraction yield increased by increasing the extraction pressure. Increasing the temperature increases the solubility of patchouli oil, to greater degradation of the bioactive component of patchouli oil. This cross-over effect between pressure and temperature also affects the density of CO_2,_ which influences the extraction time and solute component of patchouli oil. Research work by Siti Machmudah et al. [22], the recovery oil extraction was independent of pressure, although at the lowest and constant temperature. The condition of supercritical extraction in the study of variable pressure and temperature by A. Donelian et al. [16] also influenced the extraction yield. Under variable conditions, extraction could affect the density of CO_2_ and the solubilization of oil.

### 2.2. Fourier Transform Infra-Red (FT-IR)

**Figure 2:**
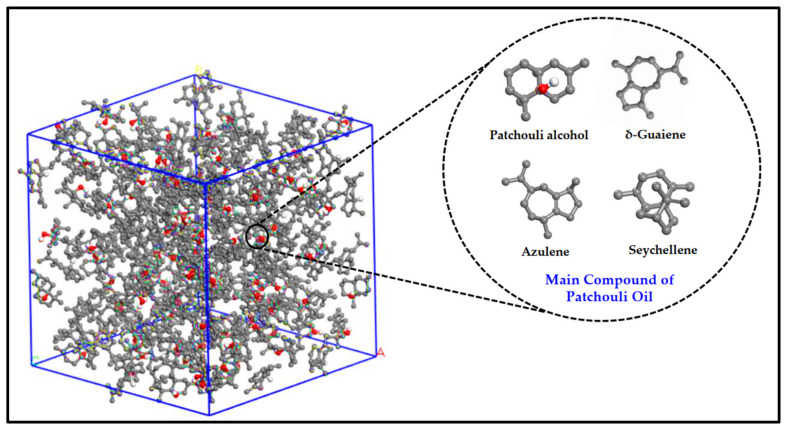
Illustration of main active compound in Patchouli extract.

**Figure 3:**
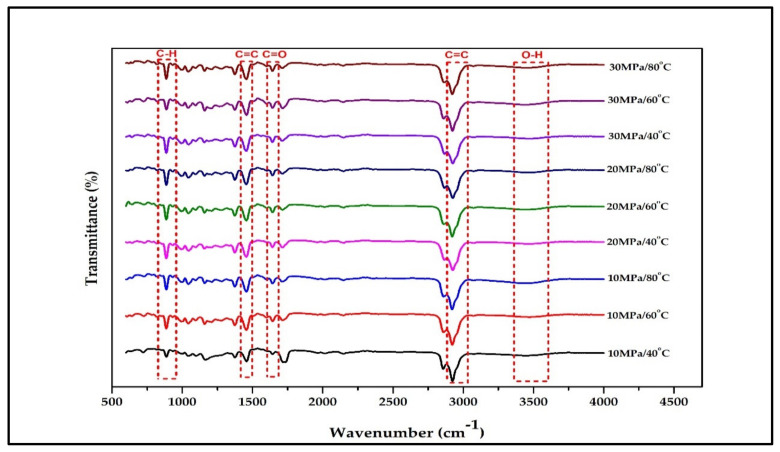
FT-IR spectrum for crude patchouli extract at different operating pressure and temperature.

Figure 2 illustrates the main active compound in the patchouli extract. There are 4 main compounds viz. Patchouli alcohol, Azulene, Seychellene and δ-Guaiene. Figure 3 illustrates the FTIR spectra for patchouli extract in a different operating pressure range (10–30 MPa) and temperature range (40–80 °C) obtained from the SC-CO_2_ fluid extraction method. Infrared (IR) spectroscopy characteristics of the functional group peaks for patchouli oil fall within the region of 500–4000 cm^−1^. It was observed that the peaks are nearly identical for all spectrums, even at varied pressures and temperatures, indicating that this approach does not affect the quality of the extract. In addition to that, there are some observable peaks of the spectrum. The broadening band at 3300–3600 cm^−1^ shows O-H stretching in alcohol [16], indicating the presence of the patchoulol compound. Another observable peak at 1635 cm^−1^ corresponds to the C=O stretching vibration of the carbonyl group in aldehyde; it showed that patchouli oil contains higher amounts of aldehyde compounds [23]. The peak at 1445 cm^−1^ can be attributed to the bending of H-C in alkane [24] and C = bending of C from aromatic rings, while the peak at 1373 cm^−1^ is a characteristic of bending of H-C in a carboxylic acid [25]. The peak represented the C-H bending vibration at 886 cm^−1^ [23].

### 2.3. GC-MS Analysis of SC-CO_2_ Extract

Analysis of the chemical properties of Patchouli extract obtained by extraction of SC-CO_2_ fluid indicates the quality of the crude patchouli extract extracted. Table 1 shows the tabulated results of the GC-MS analysis for patchouli extract extracted using SC-CO_2_ extraction in various percentage areas (%). About 14 major components in the Patchouli extract have been successfully detected using gas chromatography-mass spectrometry (GS-MS). The summary of other chemical compositions of Patchouli extract at various pressure and temperature were demonstrated in Table 2.

**Figure 4:**
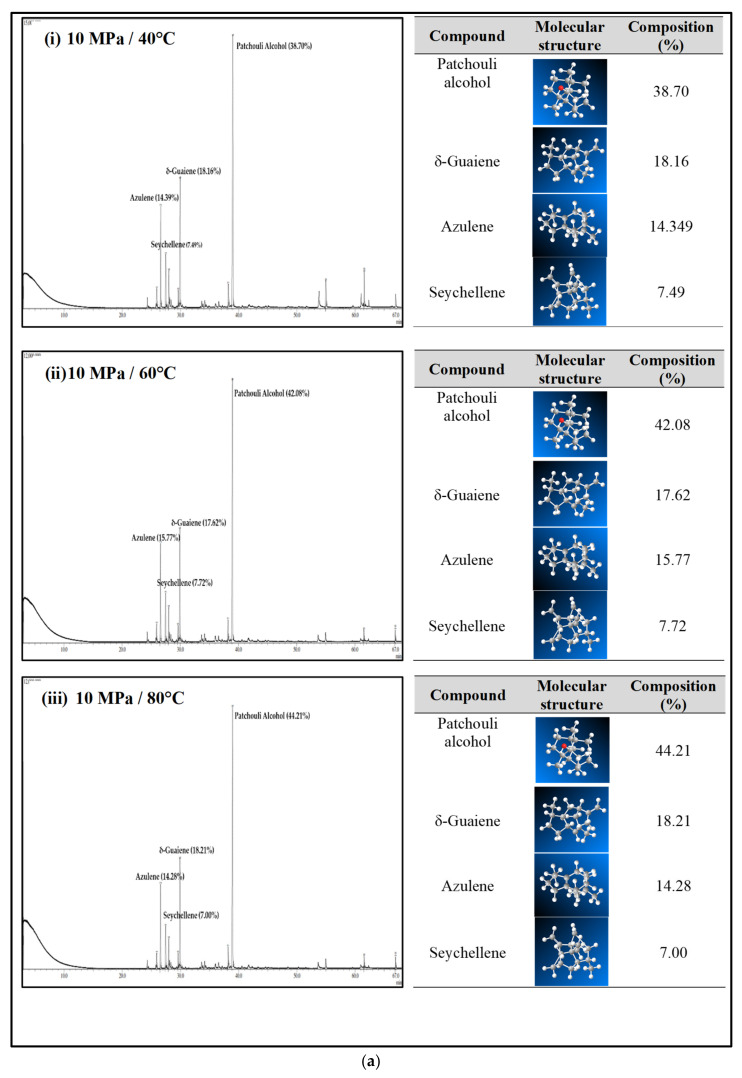

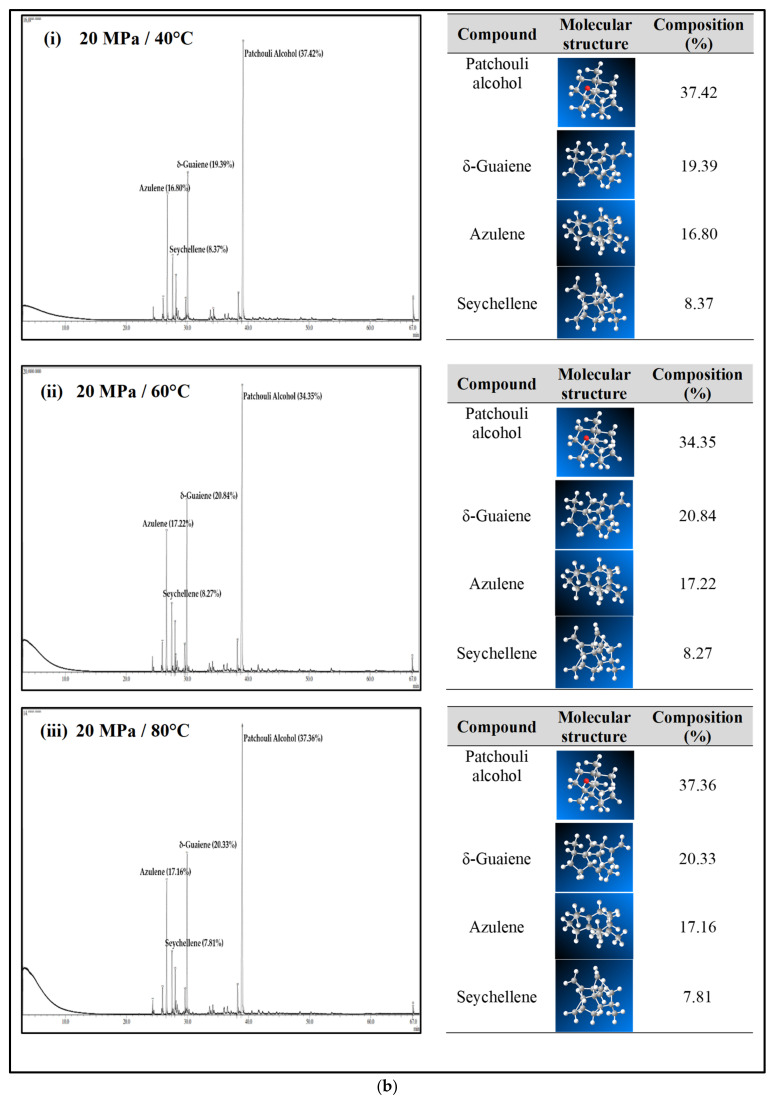

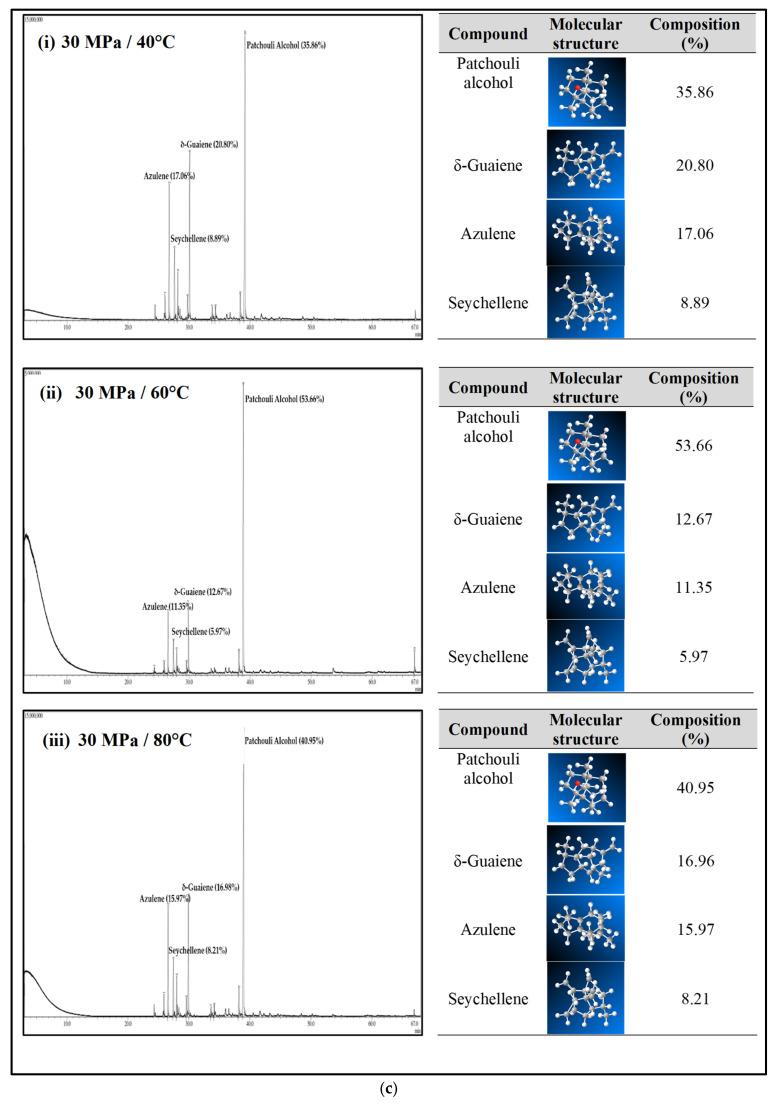
(**a**) GC-MS chromatogram of patchouli oil extracts at constant pressure (10 MPa) and temperature (40, 60 and 80 °C). (**b**) GC-MS chromatogram of patchouli oil extracts at constant pressure (20 MPa) and temperature (40, 60 and 80 °C). (**c**) GC-MS chromatogram of patchouli oil extracts at constant pressure (30 MPa) and temperature (40, 60 and 80 °C).

Figure 4a–c shows the GC-MS chromatogram of patchouli extracts at various pressures (10–30 MPa) and temperatures (40–80 °C). At pressure 10 MPa and 20 MPa, it was observed that the composition of Patchouli alcohol was highest (44.21% and 53.66%) at high temperatures (80 °C). It shows that temperature would affect extraction. An increase in temperature can improve extraction yields and speed up the process as the extraction rate increases. In Figure 4a–c, the main chemical constituents of the essential oil extracted from patchouli were found to be patchouli alcohol, Azulene, δ-Guaiene, and seychellene. The highest percentage of chemical components in Patchouli extract was Patchouli alcohol (34.35–53.66%), followed by δ-Guaiene (12.67–20.89%), Azulene (1.76–2.83%), and Seychellene (5.97–8.89%).

Based on GC peak areas of Patchouli extract, alcoholic compounds (Patchouli alcohol) represent the highest percentage in extract under various extraction conditions. The percentage of alcoholic compounds is an important parameter that determines the quality of essential oils [26]. A higher percentage of alcoholic compounds in patchouli extract has a high selling value because alcohol functional groups can easily bind to the scent. Therefore, it is widely used in the perfume industry [5]. Differences in the percentage composition of patchouli extract were observed due to differences in environmental factors, the area of origin of the plant, harvesting methods, post-harvest processing methods, extraction conditions, and oil storage [27]. The α, and δ-guaienes components of extract, and -guaienes are used in the fragrance and flavoring industries to impart spicy aromas and tastes. The α-guaiene and δ -guaiene compounds are also used in room fresheners [28]. Azulene compounds have several medical applications, such as anti-inflammatory and peptic ulcers, antineoplastic with leukemia, antidiabetes, antiretroviral with HIV-1, antimicrobial photodynamic therapy, and antifungal [29]. In addition to that, the Seychellene compound has a function as an antioxidant [30]. Table 3 shows the comparison of the components of the patchouli extract analyzed by GC-MS through different extraction methods such as steam distillation, microwave hydrodistillation, microwave air distillation, and supercritical carbon dioxide. From the result, it shows that the SC-CO_2_ extraction method gives the highest components of patchouli alcohol (38.70%) compared to steam distillation (22.70%), microwave hydrodistillation (26.32%) and microwave air hydrodistillation (25.23%). The oil extracted by steam distillation consists of the same components as SC-CO_2_, including Seychellene, α-Patchoulene, Azulene, δ-guaiene, Epiglobulol, and Patchouli alcohol. However, it shows that most of the components of patchouli oil extracted by this method (Seychellene, α-Patchoulene, Azulene, Epiglobulol, and Patchouli alcohol) give a lower percentage composition compared to SC-CO_2_. The lower value of the components was obtained due to thermal decomposition. The high temperature used in the process can deteriorate the bioactive compounds present in the extracted oil, resulting in a low composition [11]. Furthermore, some important components such as α-Patchoulene, Azulene, and Epiglobulol were not detected by GC-MS when extracted by microwave and microwave air hydrodistillation. The same applies to steam distillation; at a high extraction temperature, some volatile components may be lost [31].

## 3. Conclusions

This study has successfully extracted oily extract from Patchouli leaves, branches, and stems using SC-CO_2_ extraction methods. Under different extraction pressure and temperature conditions, the obtained oils were qualitatively characterized for chemical constituents using GC-MS and FT-IR. The results have indicated that a higher extraction yield (12.41%) and a crude patchouli oil (7.73%) were obtained at a pressure of 20 MPa and 80 °C temperature. Based on the obtained results, it may be concluded that multiparameter SC-CO_2_ extraction are able to produce a rapid and higher volume of crude patchouli oil. The GC-MS results showed that the oil consisted of 14 compounds, of which the main components are Patchoulol, Azulene, δ-Guaiene, and seychellene. Additionally, there was the presence of O-H stretching in alcohol, indicating the abundance of Patchoulol, C=O stretching vibration of carbonyl aldehyde, showing that the patchouli oil contains higher amounts of aldehyde compounds, C-H bending stretching of alkane, and C=C stretching from aromatic rings. Therefore, patchouli oil is an important plant that is endowed with various varieties of chemical compounds.

## 4. Materials and Methods

### 4.1. Materials

**Figure 5:**
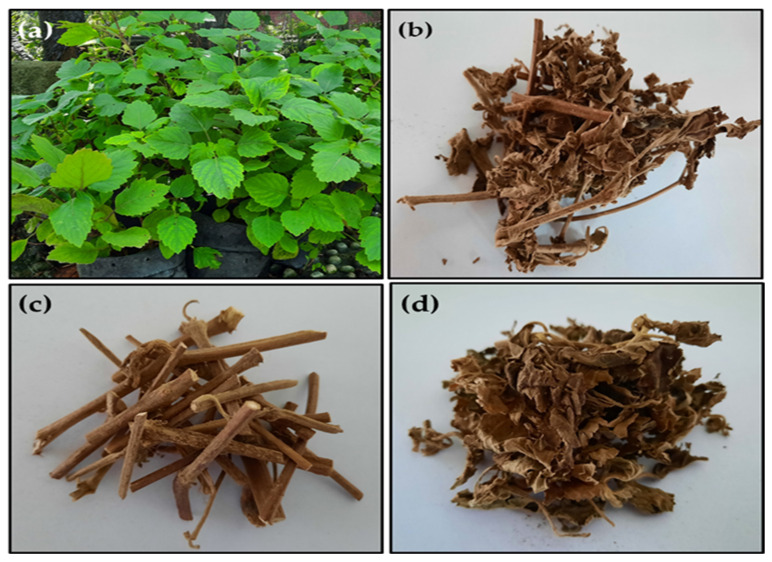
(**a**) Patchouli plant, (**b**) Dried Patchouli (leaves and branches), (**c**) Dried Patchouli branches and (**d**) Dried Patchouli leaves.

A minimum of 10 kg of dried Patchouli (mix of leaves and stems) were delivered to the School of Industrial Technology of ARC-PUIPT Nilam Aceh. Patchouli plants collected with leaves were air-dried for 24 h to prevent the development of fungi. The patchouli sample was further dried in an oven to the desired moisture content (15.61%). The dried Patchouli plant was separated into stem branches and leaves. The stem branches were cut into the size range of 3–5 cm to facilitate the extraction process. Figure 5 shows the different stages of preparation of Patchouli plant samples. The extractant (liquid CO_2_) with a purity of 99.5%, was obtained from Alpha Gas Solution Pte Ltd.

### 4.2. Sample Preparation

**Figure 6:**
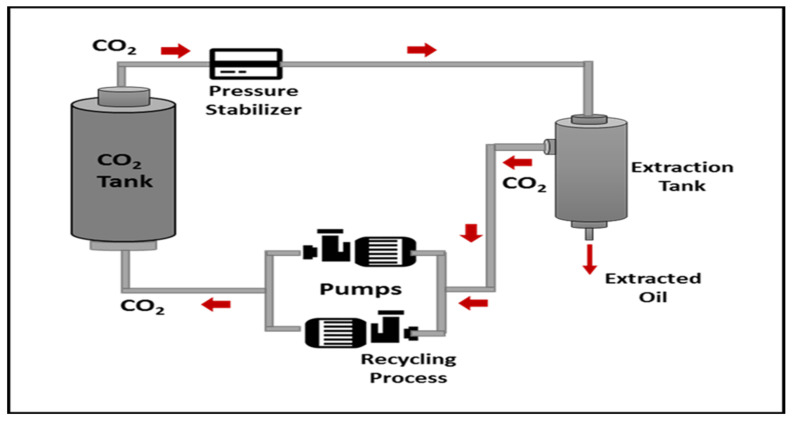
Schematic diagram of supercritical CO_2_ equipment and extraction process.

The extraction process was carried out using a supercritical CO_2_ fluid extractor (SFE) equipped with a high-pressure piston pump, stainless steel extractor tube, extracted separator, heater, and chiller. The CO_2_ gas was chilled in a chiller (at 7 °C) before being delivered to the extractor at 18 g/min. The extractor temperature was set at 40, 60 and 80 °C variously. The separator pressure was kept constant at 5 MPa, while the extractor was varied from 10, 20 and 30 MPa for 30 min to stabilize the extractor vessel before collection. The extraction yield was collected at 60 min for each extraction process. All oil samples were kept in the refrigerator until further analysis. The schematic diagram of the SC-CO_2_ extraction setup is illustrated schematically in Figure 6 [32].

### 4.3. Characterization of the Patchouli Oil

#### 4.3.1. Fourier Transform Infra-Red (FT-IR)

The FT-IR spectrum of the extracted Patchouli oil was performed using a Shimadzu instrument (Kyoto, Japan). The oil sample was analyzed using the attenuated total reflection (ATR) method with wave numbers ranging from 4000–500 cm^−1^. The wave number was used to analyze the bonding structures present in the extracted Patchouli oil by studying the position peaks in the IR spectra.

#### 4.3.2. Gas Chromatography Analysis-Mass Spectrophotometer (GC-MS)

Quantification and characterization of the constituents of the Patchouli extract were carried out by Gas Chromatography-Mass Spectrometry (Shimadzu GC-MS-QP2010) hyphenated instrument. Approximately 1 mL of patchouli extract was injected into GC-MS with the operating conditions as follows: the type of column used is Hp-5MS, the column temperature is set at 80 °C for 30 min, and the temperature speed increases about 10 °C/min up to temperature 200 °C and left for 25 min. The injector temperature is the same as the detector temperature of 310 °C. Helium gas was used as a mobile phase in the gas chromatograph. The type of electron ionization was electron impact (EI).

## Figures and Tables

**Table 1:** ANOVA test for production of crude patchouli oil by Supercritical CO_2_ process.

*Source of Variation*	*SS*	*df*	*MS*	*F*	*p-Value*	*F Crit*
Pressure (P)	76.0524	2	38.0262	62.48187	7.95 × 10^−9^	3.5545
Temperature (T)	3.6481	2	1.82405	2.99715	0.075245	3.5545
Interaction (P × T)	61.976	4	15.4941	25.45887	3.37 × 10^−7^	2.9277
Error	10.9547	18	0.60859			
Total	152.632	26				

**Table 2:** Chemical composition of Patchouli extraction oil at different temperatures and pressure.

Compounds	Comparative Chemical Composition (%)								
	10 MPa/40 °C	10 MPa/60 °C	10 MPa/80 °C	20 MPa/40 °C	20 MPa/60 °C	20 MPa/80 °C	30 MPa/40 °C	30 MPa/60 °C	30 MPa/80 °C
Β-Caryophyllene	2.45	-	2.31	2.64	3.34	3.08	2.85	1.85	2.88
Bicyclo [7.2.0]undec-4-ene, 4,11,11-trimethyl	-	2.53	-	-	-	-	-	0.98	-
Azulene, 1,2,3,4,5,6,7,8-octahydro-1,4-dimethyl	14.39	15.77	14.28	16.80	17.22	17.16	17.06	11.35	15.97
Seychellene	7.49	7.72	7.00	8.37	8.27	7.81	8.89	5.97	8.21
α-Patchoulene	4.97	5.19	4.74	5.58	5.95	5.41	5.70	4.05	5.71
Azulene, 1,2,3,4,5,6,7,8-octahydro-1,4-dimethyl	2.23	2.43	2.32	2.47	1.76	2.83	2.71	-	-
α-Guaiene	-	-	-	-	3.17	-	-	1.81	2.53
δ-Guaiene	18.16	17.62	18.21	19.39	20.84	20.33	20.80	12.67	16.98
Caryophyllene Oxide	-	-	-	1.31	-	-	1.48	-	1.47
Epiglobulol	3.01	3.06	3.19	3.43	3.65	3.57	3.09	3.95	3.95
Patchouli alcohol	38.70	42.08	44.21	37.42	34.35	37.36	35.86	53.66	40.95
Palmitate <ethyl->	3.69	-	-	-	-	-	-	-	-
(E)-9-Octadecenoic acid ethyl ester	4.90	1.77	1.93	-	-	-	-	-	-
Hexanedioic acid, bis(2-ethylhexyl) ester	-	1.82	1.80	2.59	1.44	0.89	-	3.71	-

**Table 3:** Comparison of Patchouli extract components with other extraction methods.

**No.**	**Component**	**Area (%)**			
		**Steam Distillation** [31]	**Microwave Hydrodistillation** [9]	**Microwave Air-Hydrodistillation** [10]	**Supercritical Carbon Dioxide (SC-CO_2_) [This Study]**
1	Seychellene	5.70	8.42	8.41	7.49
2	α-Patchoulene	2.88	-	11.54	4.97
3	Azulene	8.74	-	-	14.39
4	δ-guaiene	18.90	14.69	14.89	18.16
5	Epiglobulol	1.88	-	-	3.01
6	Patchouli alcohol	22.70	26.32	25.23	38.70
7	Extraction Yield	-	-	1–3%	5–12%

## Data Availability

Not applicable.

## References

[1] Jain P.L.B., Patel S.R., Desai M.A. (2022). Patchouli Oil: An Overview on Extraction Method, Composition and Biological Activities. J. Essent. Oil Res..

[2] Soh S.H., Agarwal S., Jain A., Lee L.Y., Chin S.K., Jayaraman S. (2019). Mathematical Modeling of Mass Transfer in Supercritical Fluid Extraction of Patchouli Oil. Eng. Rep..

[3] Soh S.H., Agarwal S., Jayaraman S., Tham M.T. (2018). A Study of Essential Oil Extraction and Antioxidant Activity of Patchouli (*Pogostemon cablin*) Using Supercritical Carbon Dioxide. ON12.

[4] Swamy M.K., Sinniah U.R. (2015). A Comprehensive Review on the Phytochemical Constituents and Pharmacological Activities of *Pogostemon cablin* Benth.: An Aromatic Medicinal Plant of Industrial Importance. Molecules.

[5] Ermaya D., Sari S.P., Patria A., Hidayat F., Razi F. (2019). Identification of Patchouli Oil Chemical Components as the Results on Distillation Using GC-MS. IOP Conf. Ser. Earth Environ. Sci..

[6] Xiong K., Chen Y., Shen S. (2019). Experimental Optimization and Mathematical Modeling of Supercritical Carbon Dioxide Extraction of Essential Oil from *Pogostemon cablin*. Chin. J. Chem. Eng..

[7] Aisyah Y., Anwar S.H. Physico-Chemical Properties of Patchouli Oils (Posostemon Cablin) Separated by Fractional Distillation Method. Proceedings of the 2nd Annual International Conference Syiah Kuala University & the 8th IMT-GT Uninet Biosciences Conference.

[8] Ramya H.G., Palanimuthu V., Rachna S. (2013). An Introduction to Patchouli (*Pogostemon cablin* Benth.)—A Medicinal and Aromatic Plant: It’s Importance to Mankind. Agric. Eng. Int. CIGR J..

[9] Kusuma H.S., Mahfud M. (2017). GC-MS Analysis of Essential Oil of *Pogostemon cablin* Growing in Indonesia Extracted by Microwave-Assisted Hydrodistillation. Int. Food Res. J..

[10] Kusuma H.S., Mahfud M. (2017). The Extraction of Essential Oils from Patchouli Leaves (*Pogostemon cablin* Benth) Using a Microwave Air-Hydrodistillation Method as a New Green Technique. RSC Adv..

[11] Yahya A., Yunus R.M. (2013). Influence of Sample Preparation and Extraction Time on Chemical Composition of Steam Distillation Derived Patchouli Oil. Procedia Eng..

[12] Díaz-Maroto M.C., Pérez-Coello M.S., Cabezudo M.D. (2002). Supercritical Carbon Dioxide Extraction of Volatiles from Spices: Comparison with Simultaneous Distillation-Extraction. J. Chromatogr. A.

[13] Oyekanmi A.A., Abdul Khalil H.P.S., Rahman A.A., Mistar E.M., Olaiya N.G., Alfatah T., Yahya E.B., Mariana M., Hazwan C.M., Abdullah C.K. (2021). Extracted Supercritical CO_2_ Cinnamon Oil Functional Properties Enhancement in Cellulose Nanofibre Reinforced Euchema Cottoni Biopolymer Films. J. Mater. Res. Technol..

[14] Ibáñez E., Mendiola J.A., Castro-Puyana M., Rostagno M.A., Prado J.M. (2013). Supercritical Fluid Extraction. Natural Product Extraction Principles and Applications.

[15] Soh S.H., Jain A., Lee L.Y., Chin S.K., Yin C.Y., Jayaraman S. (2021). Techno-Economic and Profitability Analysis of Extraction of Patchouli Oil Using Supercritical Carbon Dioxide. J. Clean. Prod..

[16] Donelian A., Carlson L.H.C., Lopes T.J., Machado R.A.F. (2009). Comparison of Extraction of Patchouli (*Pogostemon cablin*) Essential Oil with Supercritical CO2 and by Steam Distillation. J. Supercrit. Fluids.

[17] Soh S.H., Jain A., Lee L.Y., Jayaraman S. (2020). Optimized Extraction of Patchouli Essential Oil from *Pogostemon cablin* Benth. with Supercritical Carbon Dioxide. J. Appl. Res. Med. Aromat. Plants.

[18] Pandey S.K., Gogoi R., Bhandari S., Sarma N., Begum T., Munda S., Lal M. (2022). A Comparative Study on Chemical Composition, Pharmacological Potential and Toxicity of *Pogostemon cablin* Linn., (Patchouli) Flower and Leaf Essential Oil. J. Essent. Oil Bear. Plants.

[19] Pandey S.K., Bhandari S., Sarma N., Begum T., Munda S., Baruah J., Gogoi R., Haldar S., Lal M. (2021). Essential Oil Compositions, Pharmacological Importance and Agro Technological Practices of Patchouli (*Pogostemon cablin* Benth.): A Review. J. Essent. Oil Bear. Plants.

[20] Yang X., Zhang X., Yang S.P., Liu W.Q. (2013). Evaluation of the Antibacterial Activity of Patchouli Oil. Iran. J. Pharm. Res..

[21] Suetsugu T., Tanaka M., Iwai H., Matsubara T., Kawamoto Y., Saito C., Sasaki Y., Hoshino M., Quitain A.T., Sasaki M. (2013). Supercritical CO_2_ Extraction of Essential Oil from Kabosu (Citrus Sphaerocarpa Tanaka) Peel. Flavour.

[22] Machmudah S., Kondo M., Sasaki M., Goto M., Munemasa J., Yamagata M. (2008). Pressure effect in supercritical CO_2_ extraction of plant seeds. J. Supercrit. Fluids.

[23] Li Y.Q., Kong D.X., Wu H. (2013). Analysis and Evaluation of Essential Oil Components of Cinnamon Barks Using GC-MS and FTIR Spectroscopy. Ind. Crops Prod..

[24] Wen P., Zhu D.H., Wu H., Zong M.H., Jing Y.R., Han S.Y. (2016). Encapsulation of Cinnamon Essential Oil in Electrospun Nanofibrous Film for Active Food Packaging. Food Control.

[25] Hosseini S.F., Zandi M., Rezaei M., Farahmandghavi F. (2013). Two-Step Method for Encapsulation of Oregano Essential Oil in Chitosan Nanoparticles: Preparation, Characterization and in Vitro Release Study. Carbohydr. Polym..

[26] Muyassaroh, Daryono E.D., Hudha M.I. (2016). Determinating Patchouli Alcohol of Patchouli Oil Using Distillation Technique. Int. J. ChemTech Res..

[27] Aisyah Y., Hastuti P., Sastrohamidjojo H., Hidayat C. (2010). Increase of The Content of Patchouli Alcohol in Patchouli (*Pogostemon cablin* Benth) Oil Using Cellulose Acetate Membrane. Agritech.

[28] Souhoka F.A., Al Aziz A.Z., Nazudin N. (2020). Patchouli Oil Isolation and Identification of Chemical Components Using GC-MS. Indones. J. Chem. Res..

[29] Bakun P., Czarczynska-Goslinska B., Goslinski T., Lijewski S. (2021). In Vitro and in Vivo Biological Activities of Azulene Derivatives with Potential Applications in Medicine. Med. Chem. Res..

[30] Raharjo S.J., Mahdi C., Nurdiana N., Kikuchi T., Fatchiyah F. (2015). In Vitro and In Silico: Selectivities of Seychellene Compound as Candidate Cyclooxygenase Isoenzyme Inhibitor on Pre-Osteoblast Cells. Curr. Enzym. Inhib..

[31] Hasbay I., Galanakis C.M., Galanakis C. (2018). 7—Recovery Technologies and Encapsulation Techniques. Polyphenols: Properties, Recovery and Applications.

[32] Nasution H., Yahya E.B., Abdul Khalil H.P.S., Shaah M.A., Suriani A.B., Mohamed A., Alfatah T., Abdullah C.K. (2022). Extraction and Isolation of Cellulose Nanofibers from Carpet Wastes Using Supercritical Carbon Dioxide Approach. Polymers.

